# An updated analysis of turning point, duration and attack rate of COVID-19 outbreaks in major Western countries with data of daily new cases

**DOI:** 10.1016/j.dib.2020.105830

**Published:** 2020-06-11

**Authors:** Wei wei, Xiaolei Zhang

**Affiliations:** a School of Medicine, Pingdingshan University, Pingdingshan, Henan, China; b Pan-Asia Business School, Yunnan Normal University, Kunming, China

**Keywords:** Daily new cases, Governments’ interventions, Seven-day lag, Segmentation, Statistical analysis, Dynamic prediction

## Abstract

As coronavirus spreads around the world, the study of its effects is of great practical significance. We collated data on daily new cases of the COVID-19 outbreaks in the six Western countries of the Group of Seven and the dates of governments’ interventions. We studied the periods before and after the dates of major governments’ interventions integrally based on a segmented Poisson model. The relevant results are published in the paper of “Predicting turning point, duration and attack rate of COVID - 19 outbreaks in major Western countries” [1]. Our method can be used to update prediction daily as COVID-19 outbreaks evolve. In this article, we illustrate an updated analysis with our method to facilitate reproducibility. Both datasets used and updated are provided.

Specifications table

**Table:** 

**Subject**	Infectious Diseases
**Specific subject area**	Segmented Poisson model applied to predict turning point, duration and attack rate of COVID-19 outbreaks
**Type of data**	Table Figure
**How data were acquired**	The data on daily new confirmed cases of COVID-19 were taken from Wind Database live updates, and the data are up to May 8,2020. The used and updated data were built as a time-series database by Excel 2017. The relevant covariates incorporating governments’ interventions in the segmented Poisson model were established for analysis using Rstudio software.
**Data format**	Raw
**Parameters for data collection**	In the process of data collection, we considered the availability and authority of data and chose Wind Database live updates as the data source. At the same time, we looked up the dates of the state interventions, taking into account the characteristics of the data structure.
**Description of data collection**	The data of daily new cases form Wind Database is directly exported to Excel.
**Data source location**	Country: Canada, France, Germany, Italy, UK and USA
**Data accessibility**	With the article
**Related research article**	Predicting turning point, duration and attack rate of COVID-19 outbreaks in major Western countries XiaoleiZhang, lecturer RenjunMa, Professor LinWang, Professor Chaos, Solitons & Fractals https://doi.org/10.1016/j.chaos.2020.109829

## Value of the Data

Data of daily new cases are useful because they can be used to predict COVID-19 outbreaks, while dates of governments’ interventions are important for accurate analysis of changes in COVID-19 outbreaks. These two parts of data are of practical significance for the analysis and intervention of COVID-19. At the same time, the updated data can more accurately track and analyze the development of COVID-19.
At present, these data are being updated daily. The analysis of new data with our model can be updated daily to provide dynamic information about COVID-19 development to relevant departments and society. The updated data has more comprehensive information and more accurate analysis of the epidemic. On the one hand, it shows the dynamic updating characteristics of the method; on the other hand, it is of more practical significance for the later analysis of COVID-19.Researchers can use this data to replicate the models in the previous articles, and institutions involved in public health and infection control can use the updated data to track COVID-19 in real time.

## 1 Data description

**Fig. 1:**
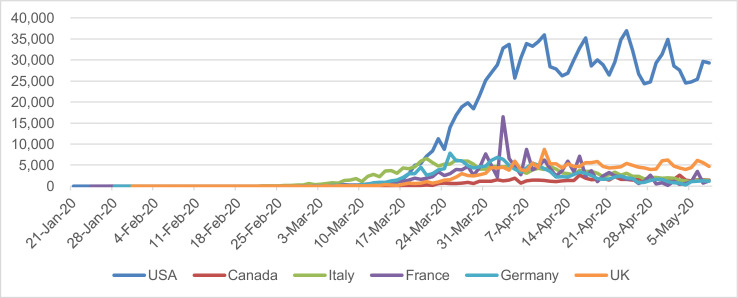
Daily new confirmed cases in each country.

The data on daily new confirmed cases of COVID-19 in these countries we used were taken from the Wind Database and from the webpage on US and Canada COVID-19 live updates [2]. In order to analyse the data more accurately, we collected the dates of major government's interventions, such as governments’ enforcement of stay-at-home advises/orders, social distancing, lockdowns, and quarantines against COVID-19, and processed the data in sections. Considering the delayed effect of government interventions, we chose 7 days after the intervention date as the segmentation point. Table 1 shows the dates of government's intervention of each country and Fig. 1 shows the data of daily new confirmed cases in each country.

**Table 1:** Dates of government's interventions.

USA	Canada	Italy	France	Germany	UK
16-March	16-March	6-March	13-March	22-March	23-March

## 2 Experimental design, materials, and methods

Through the observation of the data, we found that the change of the epidemic can be characterized by the combination of the power law with the exponential law for daily new cases [3]. Previous studies also show that the data conform to the exponential law [4]. According to epidemiological investigation, the incubation period of covid-19 is 3–7 days. After novel coronavirus infection, the patient may develop symptoms such as fever, dry cough and pneumonia for about 3 to 7 days. Considering the incubation time of the virus, we delayed the data by 7 days to take into account the changes in the data after the government's interventions.

To take major government's interventions into account, we incorporated segmentation into Poisson model [5]. The specific steps for segmentation include two parts. The first step is to divide the data into two parts. The first part of the data will be seven-day lag, depending on the date of government intervention, and the data for seven days after the government intervention will be included in the second part. The second step is to construct the corresponding covariables for the segmented data, so that the data before and after the segmentation become a whole for the Poisson model.

**Fig. 2:**
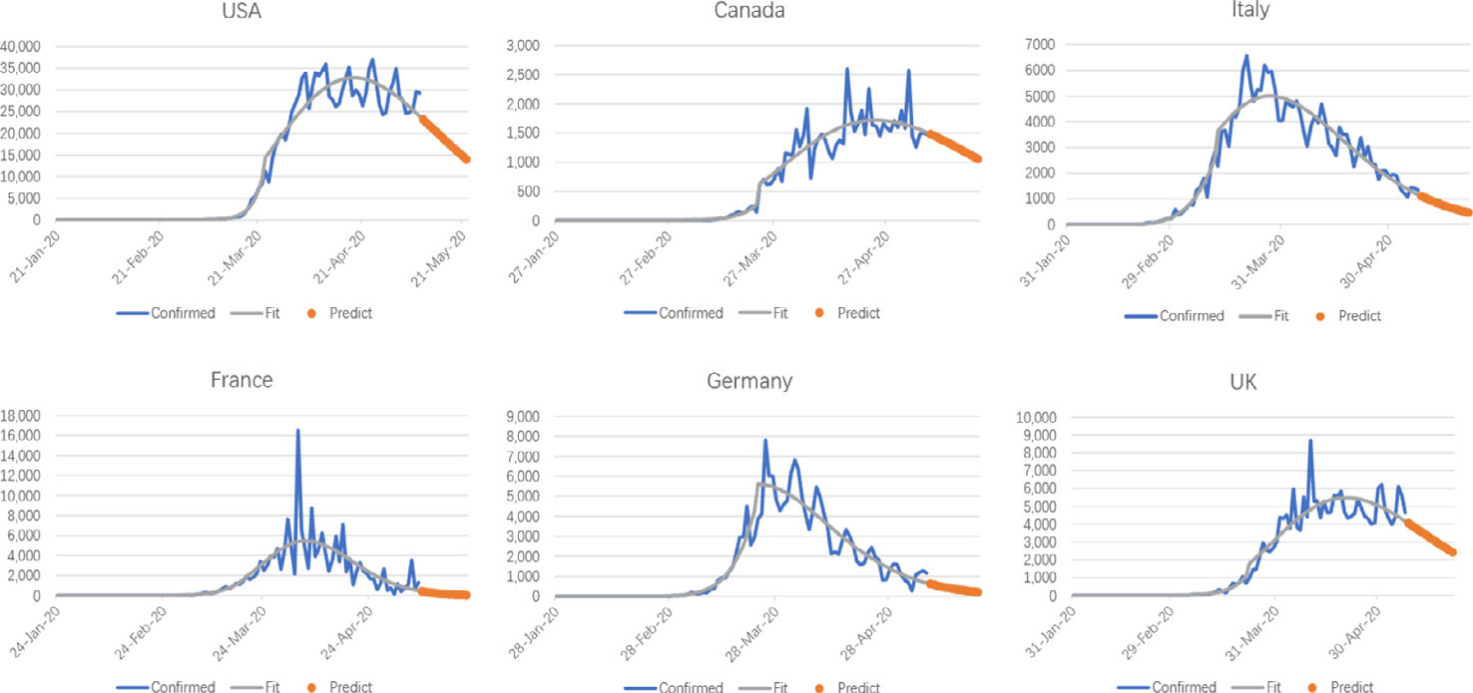
Daily new confirmed cases, fitted and predictive values in each country.

In order to model the segmented data as a whole, we created six covariates, three each for the periods before and after major government's interventions. Among them, X_1_, X_2_ and X_3_ were set as were 1, t and log(t) before the segmentation and all 0 after the segmentation; X_4_, X_5_ and X_6_ were all set as 0 s before the segmentation and 1, t and log(t) after the segmentation. In this way, the segmented data can be analysed using a unified model for parameter estimation. The updated prediction of turning point, duration, final size and attack rate of COVID-19 based on data up to May 8,2020 are shown in Fig. 2 and Table 2.

**Table 2:** Updated prediction of turning point, duration, final size and attack rate of COVID-19.

Country	Turning point(Range)	Final size	Duration	Attack rate
USA	Apr.18(Apr.16–Apr.20)	1,817,322	Jan.21–Sep.16	0.55%
Canada	Apr.24(May.02–Apr.20)	108,641	Jan.27–Sep.12	0.29%
Italy	Mar.28(Mar.25–Mar30)	233,301	Jan.31–Aug.01	0.39%
France	Apr.05(Apr.04–Apr.07)	179,204	Jan.24–Jun.13	0.27%
Germany	Mar.23(Mar.19–Mar27)	178,723	Jan.28–Jul.13	0.21%
UK	Apr.21(Apr.17–Apr.25)	297,167	Jan.31–Aug.27	0.44%

In the previous published articles, we used the data until April 9, and the updated data until May 8.When the trend of the epidemic changes is more clearer, the result of our model is more accurate in the analysis of COVID-19.Therefore, based on the updated data, the dynamic update method is used to estimate the relevant data more accurately.

## Declaration of Competing Interest

The authors declare that they have no known competing financial interests or personal relationships which have, or could be perceived to have, influenced the work reported in this article.

## Appendix Supplementary materials

Image, application 1

Image, application 2

Image, application 3
